# A Systematic Review of Nano- and Microplastic (NMP) Influence on the Bioaccumulation of Environmental Contaminants: Part I—Soil Organisms

**DOI:** 10.3390/toxics11020154

**Published:** 2023-02-07

**Authors:** Paula S. Tourinho, Susana Loureiro, Maria D. Pavlaki, Klará Anna Mocová, Fabianne Ribeiro

**Affiliations:** 1 Department of Environmental Chemistry, Faculty of Environmental Technology, University of Chemistry and Technology Prague, Technická 5, 166 28 Prague, Czech Republic; 2 Department of Biology and CESAM, University of Aveiro, Campus Universitário de Santiago, 3810-193 Aveiro, Portugal

**Keywords:** literature review, bioavailability, combined exposure, plastic pollution

## Abstract

Nano- and microplastics (NMPs) are a group of contaminants that cause concern due to their abundance in the environment, high persistence, and interaction with other contaminants. This review aims to understand the role of NMP in the bioaccumulation of environmental contaminants. For that, a comprehensive literature search was conducted to identify publications that compared the uptake of contaminants in the presence and absence of NMP. In this part I, twenty-eight publications of the terrestrial compartment were analyzed. Two main taxonomic groups were studied, namely, earthworms and terrestrial plants. In earthworms, most studies observed an increase in the bioaccumulation of the contaminants, while in plants, most studies observed a decrease in the bioaccumulation. Changes in bioavailable fractions of contaminants due to NMP presence was the main reason pointed out by the authors for their outcomes. Moreover, biological aspects were also found to be important in defining how NMPs affect bioaccumulation. Dermal damage and changes in contaminant-degrading bacteria in the gut of earthworms caused an increase in bioaccumulation, and root pore blockage was a common reason for the decrease in the bioaccumulation of contaminants in plants. Nevertheless, such effects were mainly observed at high, unrealistic NMP concentrations. Finally, knowledge gaps were identified, and the limitations of this systematic review were presented.

## 1. Introduction

Nano- and microplastics (NMPs) are one of the main pollutants of concern nowadays for several reasons. Firstly, NMPs are abundantly found in the environment, and reports of their presence have been made worldwide [1]. This is, in great part, a reflection of high waste generation (e.g., as much as 6300 million metric tons of plastic have been produced since the 1950s [2]). Most plastic waste has land-based origins; however, it can be transported and/or retained in different compartments [3]. Moreover, NMP abundance in the environment has been estimated to continue increasing in the following decades [4]. Besides the undoubted presence of NMPs in the environment, other reasons for concern are linked to possible toxicological effects on biota and humans [5], contribution to climate change by releasing greenhouse gases [6], and interactions with other organic and inorganic contaminants [7]. The later impact of NMP has raised major questions in the scientific community concerning the role of NMP as a vector of contaminants [8,9,10].

There is a great body of literature on NMP interaction with contaminants, especially via sorption/desorption processes [11,12]. The sorption/desorption of contaminants on NMP depends on chemical affinity (related to their physicochemical characteristics) and other factors such as their concentrations, type of medium, and time. Chemical analysis revealed high concentrations of contaminants in NMP collected in the environment [13,14,15,16]. In some cases, the contaminant concentration in NMP may even exceed that of the surrounding environment [13]. The levels of contamination in NMP also increased with time, indicating that the prolonged presence of NMP may increase the accumulation of contaminants [15]. One of the most important aspects of these interactions is that NMP may change the available fractions of the contaminants [17,18,19] and, therefore, directly impact bioaccumulation.

Bioaccumulation, hereby defined as the increase in contaminant levels in an organism’s body, is the first step of possible toxic effects. It results from the net uptake and elimination rates of the contaminant and is a critical process, defining the potential of a contaminant to trigger adverse effects. The uptake of contaminants can occur via different routes in the environmental medium (water, soil, and sediment), including ingestion and dermal uptake in biota and rhizofiltration in plants. NMP may change the processes involved in bioaccumulation. Firstly, NMP may be a new route of exposure to environmental contaminants, for example, via the ingestion of contaminated NMP. Moreover, NMP may affect the internal processes of uptake/elimination in the organisms, affecting the overall bioaccessibility of contaminants. Considering the increasing levels of NMP in the environment and their interaction with contaminants, it is crucial to understand if NMPs are likely to affect the bioaccumulation of contaminants. 

The number of studies on the joint exposure of NMP and contaminants has been gradually increasing over the years [7,11]. While there are many scientific publications and review articles on the (eco)toxicological effects of their joint exposure, the consequence of bioaccumulation is less explored. Therefore, a review paper focused on the effects of bioaccumulation alone may bring valuable insights to help fill this gap. 

This paper covers a systematic scoping review of the literature, concerning the impact of NMP exposures on the bioaccumulation of toxic compounds in terrestrial organisms. Following the Preferred Reporting Items for Systematic Reviews and Meta-Analyses (PRISMA), this review aims to find evidence on the interaction of NMP–chemicals–bioaccumulation and identify knowledge gaps on this topic. We aim to answer the question: how does NMP’s presence affect the bioaccumulation of contaminants? For that, only publications that compared treatments with or without NMP were included. After the literature selection, the articles were separated into three main groups to make the systematic review as comprehensive as possible: namely, terrestrial, freshwater, and marine. This review focuses on the terrestrial publications retrieved from our literature search. Soils are an important sink for NMP [20], and more research is needed to determine the impacts of NMP in the terrestrial environment [1,11].

## 2. Materials and Methods

### 2.1. Literature Search

This review followed the guidelines from the Preferred Reporting Items for Systematic Reviews and Meta-Analyses (PRISMA-) [21]. A comprehensive search was performed in Web of Science and Scopus databases, using the algorithm: (microplastic* OR nanoplastic*) AND ((toxic compound* OR pollutant* OR contaminant* OR hydrophobic organic contaminant* OR persistent organic contaminant* OR metals) (bioaccumulation OR accumulation OR desorption)) AND (environment* OR ecotoxic*). The search retrieved 995 and 2606 articles from Web of Science and Scopus, respectively. After an initial check, 422 duplicates were found. Afterward, the title and abstract screening of 3179 articles was conducted. 

### 2.2. Literature Selection and Data Collection

An inclusion criterion was used during the title and abstract screening. Only original research articles published in English were considered. Other publications, such as reviews, book or book chapters, and conference papers, were not included. Moreover, studies that did not explicitly indicate the joint exposure to nano/microplastics and contaminants or the use of environmentally important species (i.e., animal models used in medical studies) were excluded.

The studies included in the final selection had their data extracted into a table in excel. Information on the study characteristics included the exposure route (soil or hydroponic medium) and spiking procedure. Three spiking procedures were included: NMP and contaminant added to the medium at the beginning of the experiment (freshly spiked), NMP and contaminant added to the medium and incubated before exposure (pre-incubation), or NMP contaminated with the contaminant in water before being added to the exposure medium (pre-contamination). The characteristics of NMP were included (size, form, and polymer type). The studies were further classified, using particle size, as microplastic (MP) experiments, for particles between 1 µm and 5 mm, and nanoplastic (NP) experiments, for particles below 1 µm [22,23]. Moreover, information on the contaminants (name and chemical group), organisms (species and taxonomic group), and the outcomes of bioaccumulation were also included.

## 3. Results

### 3.1. Literature Search and Selection

**Figure 1:**
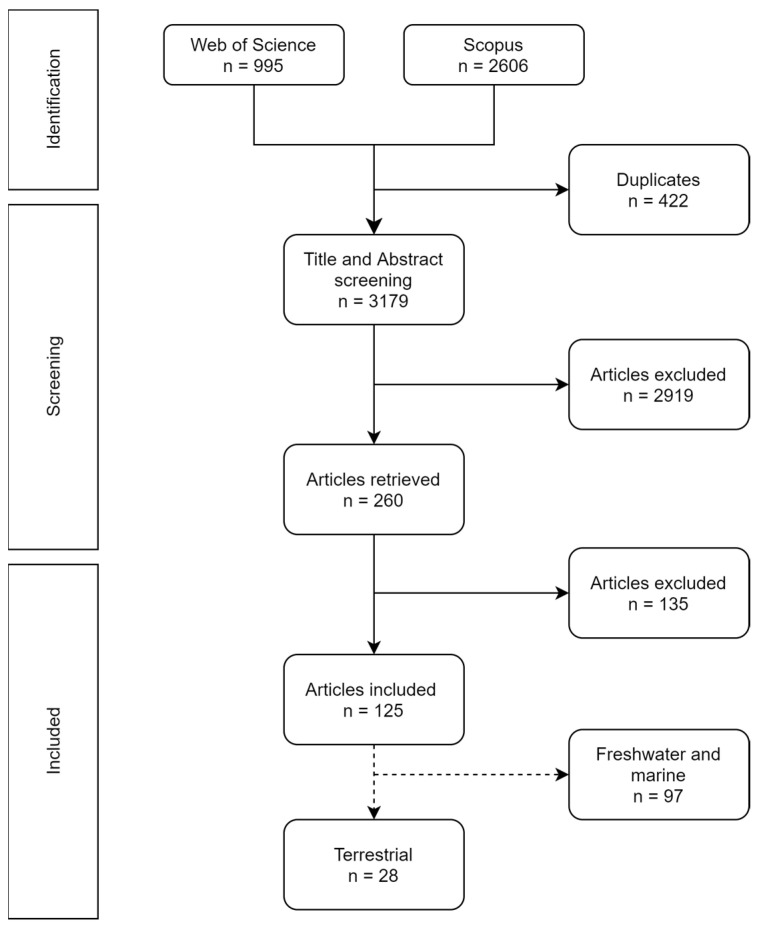
Flow diagram of the study selection process.

The number of publications in each phase of the literature search and selection can be found in Figure 1. After manual title and abstract screening of 3179 articles, a total of 260 articles were identified as relevant and had the full text analyzed. One hundred and thirty-five further articles were excluded due to (1) a lack of treatments exposed to contaminants alone to allow comparison; (2) plastic particles were too large to be considered as microplastics; (3) desorption studies (e.g., desorption of contaminants under gut-simulated conditions); (4) NMP was added only in the depuration phase; and (5) field studies. From the 126 publications included, 28 were classified as terrestrial and included in this systematic review (Table S1).

Some publications used more than one type of NMP or test species. Therefore, we found it necessary to distinguish between the number of publications and the number of experiments. For example, a publication with two types of NMP (i.e., different sizes, shapes, and polymer types) was counted as two experiments. The 28 publications resulted in a total of 36 experiments. 

### 3.2. Characteristics of Selected Studies

**Figure 2:**
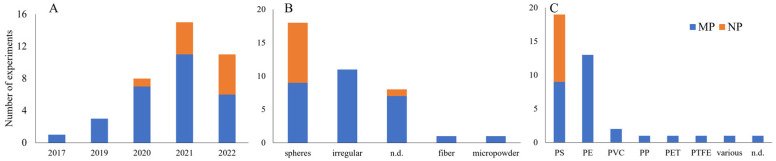
(**A**) Number of experiments per year, (**B**) shape, and (**C**) polymer type of microplastics (MP) and nanoplastics (NP) from the 28 publications selected. N.d.: not determined; PS: polystyrene; PE: polyethylene; PVC: polyvinyl chloride; PP: polypropylene; PET: polyethylene terephthalate; and PTFE: polytetrafluoroethylene.

The selected studies were published between 2017 and 2022, with almost 90% published from 2020 to 2022 (Figure 2). Microplastics (herein defined as ≥1 µm to <5 mm) and nanoplastics (<1 µm) were used in 73.7 and 26.3% of the experiments. Nevertheless, this difference is declining, as the number of MP experiments went from 7 times higher than NP in 2020 to only 1.2 times higher in 2022 (Figure 2). Regarding NMP shape, spheres/beads were used in 44.7% of the experiments, irregular NMP in 28.9%, and fiber and micropowder in 2.6% each (Figure 2). Seven studies (21.1%) provided no information on particle shape. Polystyrene (PS) (50%) and polyethylene (PE) (31.6%) were the most studied polymer type, followed by polyvinyl chloride (PVC) (5.3%) (Figure 2). Polyethylene terephthalate (PET), polypropylene (PP), and polytetrafluoroethylene (PTFE) were used in one experiment each (2.6%). One study (2.6%) used a mixture of polymer particles collected from agricultural soils, and another did not inform the NMP type.

The contaminants were classified into major chemical groups, considering both chemical properties and use. Metals were the most studied group (51.2%), followed by polycyclic aromatic hydrocarbons (PAHs) (18.6%), polychlorinated biphenyls (PCBs) (9.3%), and nanoparticles (4.7%). Pesticides, phthalates, and per- and polyfluoroalkyl substances (PFASs) were used in 4.7% of the experiments each, and pharmaceuticals and personal care products (PPCPs) in 2.3% each.

**Figure 3:**
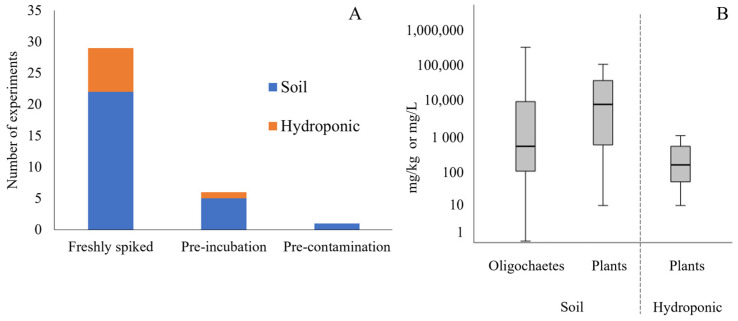
(**A**) Number of experiments in soil and hydroponic mediums using different spiking procedures. (**B**) Box plot of nano- and microplastic (NMP) concentration in soil (mg NMP/kg) and hydroponic (mg NMP/L) exposures.

In most experiments (80.6%), organisms were exposed to soil or hydroponic medium freshly spiked with NMP (Figure 3). Pre-incubation (i.e., medium incubated with NMP and contaminant before exposure) were applied in 16.7% of the experiments, and the incubation period was from 2 to 14 days. Pre-contamination of NMP before being added to the exposure medium was found in 2.8% of the experiments. The NMP concentration ranged from 0.01 to 300,000 mg/kg in soils and from 10 to 1000 mg/L in hydroponic exposure (Figure 3). Oligochaetes (earthworms and potworms) and terrestrial plants were the only two taxonomic groups studied, appearing in 15 and 13 publications, respectively. These studies are described in detail in the following sections.

### 3.3. Studies with Earthworms and Potworms 

**Figure 4:**
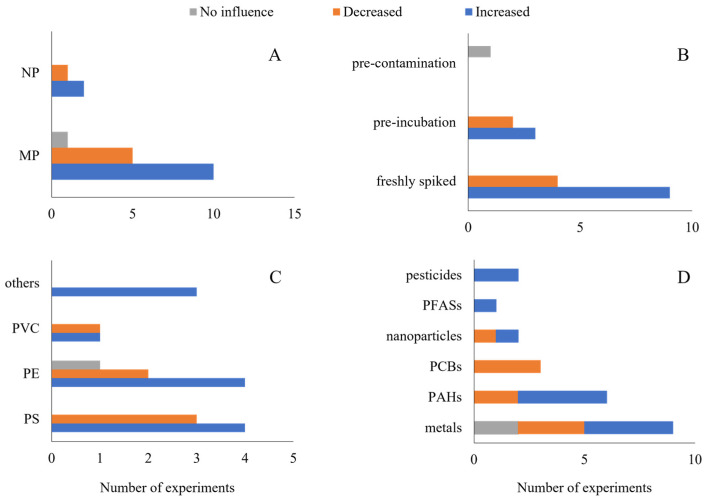
Outcome on the influence of nano- and microplastics (NMPs) on the bioaccumulation of contaminants in earthworms. Studies’ characteristics are shown as (**A**) particle size, (**B**) spiking procedure, (**C**) type of polymer, and (**D**) type of contaminant. NPs: nanoplastics; MPs: microplastics; PVC: polyvinyl chloride; PE: polyethylene; PS: polystyrene; PFASs: per- and polyfluoroalkyl substances; PCBs: polychlorinated biphenyls; and PAHs: polycyclic aromatic hydrocarbons.

Soil was the exposure medium used in all studies with oligochaetes. The earthworms *Eisenia fetida* and *E. andrei* were used in 90% of the experiments, while *Lumbricus terrestris* and *Metaphire californica* were used in 5% of the experiments each. Regarding the bioaccumulation of contaminants, diverged outcomes were observed. The characteristics of NMP and contaminants are shown in Figure 4.

More than half of the experiments (52.2%) observed an increase in the bioaccumulation of contaminants when NMPs were added to the soils. Microplastics and nanoplastics, composed of different polymer types, were either pre-incubated with contaminants or were freshly spiked to the soils. The presence of NMP increased the bioaccumulation of both metals and PAHs in 17.4% of experiments, pesticides in 8.7%, and nanoparticles and PFASs in 4.3% each. 

A decrease in bioaccumulation was observed in 39.1% of the experiments with earthworms. MP and NP, composed of PS, PE, and PVC, were either pre-incubated or freshly spiked. Metals’ and PCBs’ bioaccumulation decreased in 13% of experiments each, PAHs in 8.7%, and nanoparticles in 4.3%. 

One study found that NMP did not influence the bioaccumulation of Zn in earthworms [24]. It was also the only study where MPs were pre-contaminated.

The potworm, *Enchytraeus crypticus*, was only used in one experiment, which did not find differences in bioaccumulation of Ag, as nanoparticles or AgNO_3_, when exposed along with PET MP [25].

### 3.4. Studies with Plants

**Figure 5:**
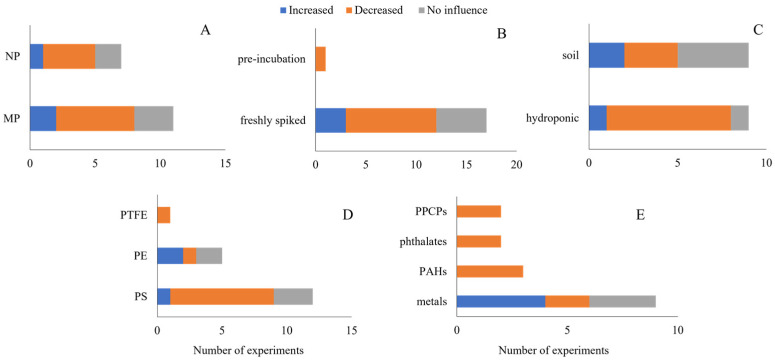
Outcome on the influence of NMP on the bioaccumulation of contaminants in terrestrial plants. Studies’ characteristics are shown as (**A**) particle size, (**B**) spiking procedure, (**C**) exposure medium, (**D**) type of polymer, and (**E**) type of contaminant. NPs: nanoplastics; MPs: microplastics; PTFE: polytetrafluoroethylene; PE: polyethylene; PS: polystyrene; PPCPs: pharmaceuticals and personal care products; and PAHs: polycyclic aromatic hydrocarbons.

Eight terrestrial plants were tested in total, namely, *Brassica napus, Glycine max, Pisum sativum, Lactuca sativa, Lolium perenne, Oryza sativa, Triticum aestivum,* and *Zea mays*. The characteristics of NMP, contaminants, and experiments are found in Figure 5. Most experiments (56.3%) found that NMP decreased the bioaccumulation of contaminants (Figure 5). NMPs were composed of PS, PE, and PTFE, which were freshly spiked or pre-incubated. In the experiments, water was the route of exposure in plants, with NMP concentrations ranging from 0.04 to 100 mg/L. PAH bioaccumulation decreased in 18.8% of the experiments, while metals’, phthalates’, and PPCPs’ bioaccumulation decreased in 12.5% of the experiments. 

The bioaccumulation of metals was the only one found to increase in the presence of NMP, representing 18.8% of the experiments with plants. All experiments were conducted in a freshly spiked medium. No influence of NMP on metal bioaccumulation was observed in 25% of the experiments.

## 4. Discussion

### 4.1. NMPs Are Most Likely to Affect Bioaccumulation

This systematic review aimed to answer one central question: whether NMP affected the bioaccumulation of contaminants by comparing the co-exposure of contaminants and NMP versus the single exposure to the contaminant. For that, only publications that had these two treatments (i.e., contaminant with and without NMP) under the same conditions were included. Our results showed that NMP affected bioaccumulation in over 91% and 76% of the studies using earthworms and plants, respectively. 

NMP concentration seems to play an important role, as many studies observed that effects on bioaccumulation were increased or decreased with increasing NMP levels. More specifically, contaminant bioaccumulation decreased in seven experiments [26,27,28,29,30,31] and increased in three experiments [32,33,34] when NMP levels were increased. Nevertheless, these effects on bioaccumulation occurred at relatively high NMP levels: 500 mg/kg NMP when co-exposed with Ni [27] and ≥1000 mg/kg NMP co-exposed with metals, PAHs, pesticides, and PFASs [28,29,32,33,34]. An exception was the study by Yang et al. [30], where Pb and Ni levels in earthworms increased at 10 mg NMP/kg. 

Several reasons were attributed to increased contaminant bioaccumulation in earthworms and plants. One common explanation given by the authors was that NMP presence could increase the bioavailability of contaminants [27,33,35,36]. However, this was only observed for metals in earthworm and plant studies. By changing soil properties, NMP can decrease the adsorption capability of metals to the soil, increasing their bioavailable fraction [27,36]. Wang et al. [36] observed that NMP presence decreased soil pH and cation exchange capacity while increasing dissolved organic matter, promoting Cd desorption from the soil. 

Other explanations for the bioaccumulation increase were related to the biological characteristics or traits of the studied organisms. In earthworms, the ingestion of contaminated NMP caused an increase in contaminant levels [32,34,37,38,39]. NMP concentration and size are important factors when ingestion is the mainly via of exposure to contaminants. NMP uptake by earthworms increases with increasing soil concentration [40], and it must be small enough to enter the oral cavity. Moreover, direct adverse effects on organisms caused by NMP could explain the bioaccumulation increase. NMP caused skin damage in earthworms, increasing phenanthrene’s dermal uptake and lowering phenanthrene-degrading bacteria in the earthworms’ gut [41]. In plants, the internalization of 200 nm NP in roots could also explain the increase in contaminant uptake [42]. Nevertheless, the size of NMP for internalization via root uptake varies among studies. Li et al. [43] observed that 200 nm PS could translocate in wheat via the vascular system, while Taylor et al. [44] did not observe any internalization of 40 nm PS in the same plant species.

The presence of NMP could also decrease the bioaccumulation of contaminants, as shown by many studies. Similarly, NMP affected the bioavailable fraction of contaminants, but in this case, decreased the bioaccumulation in both earthworms [25,29,45,46] and plants [26,28,47,48]. This was observed for metals and organic compounds, such as phenanthrene and PCBs [28,29,46]. In plants, where most of the outcomes indicated a decrease in bioaccumulation, physical and physiological effects could also explain such results. The blockage of root pore by NMP [49,50] and a decrease in root system activity [50] has been suggested to decrease contaminant uptake in plants.

The studies, which found that NMP did not affect bioaccumulation, were all conducted with metals in earthworms and plants. This could be explained by the fact that metals normally have lower adsorption coefficients for NMP than hydrophobic compounds [51]. An exception was one study with Ag NP in potworms, where the large size of the MP might have resulted in no ingestion by the potworms. It might be that NMP presence has a lesser influence on metal bioaccumulation because of the lower adsorption capacity of metals. Moreover, these studies used, in general, lower NMP concentrations when compared to most of the other studies compared herein. In soil, NMP concentration ranged from 20 to 4500 mg NMP/kg, and 10 mg NMP/L was used in a hydroponic exposure. Exceptions were the studies by Wang et al. [52] and [53], where NMP concentrations ranged from 1000 to 100,000 mg NMP/kg; however, no effect on Cd accumulation in plants was observed. The low Cd concentration of 5 mg/kg could also be related to this outcome. The authors believe that, although PS MP (1000 and 10,000 mg NMP/kg) increased the available Cd fractions, other physiological effects on plants prevented higher Cd bioaccumulation (e.g., lower water and Cd availability caused by MP and decreased root biomass caused by Cd). However, these speculations failed to explain the outcomes of bioaccumulation. Firstly, if root biomass decreased due to Cd uptake, the concentration of Cd in the root would increase and not the opposite. Secondly, the bioavailable Cd fraction decreased at the highest PE concentration (10% or 100,000 mg NMP/kg), but no difference in Cd bioaccumulation was observed between plants exposed to the presence or absence of MP. 

**Figure 6:**
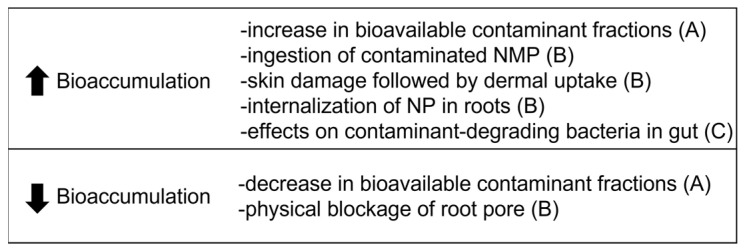
Overview of the mechanisms involved in the bioaccumulation of contaminants in the presence of nano- microplastics (NMP). Refer to the text for an explanation of the letters A, B, and C.

An overview of the mechanisms affecting the bioaccumulation of contaminants caused by NMP presence is given in Figure 6. Most mechanisms presented by the authors are related to the chemical and biological bioavailability of contaminants. These include (A) the adsorption/desorption process of contaminants to NMP; (B) the exposure route (oral and dermal contaminant uptake in earthworms and root uptake in plants); and (C) the metabolization of contaminants. 

The role of NMP as a vector of contaminants is intrinsically related to biological and chemical processes, as follows: adsorption of contaminant from medium to NMP, uptake of contaminated NMP by the organism by the organism, desorption of the contaminant in the body or rhizosphere followed by absorption into the vascular system or transfer of contaminated NMP into the vascular system. 

Considering the contaminants’ concentration in the environmental matrix is higher than in the NMP, the transfer of the contaminant from the medium to the NMP occurs. The adsorption is highly dependent not only on the chemical properties of NMP and contaminant but also on several environmental factors and may be a rather complex process [11]. While some factors are well known to affect the adsorption of contaminants to NMP (e.g., soil pH, organic content), other factors are less explored. For example, microorganisms can have direct and indirect effects on adsorption and, consequently, on contaminants’ bioaccumulation. Wang et al. [36] observed that the presence of NMP and contaminants changed the soil’s microbial composition, which increased the available fractions of the contaminant. Regardless of the complexity of the process, most studies in this review concluded that bioaccumulation was increased due to the adsorption of the contaminant, followed by the NMP uptake in earthworms and plants. 

However, whether the NMP adsorbed with contaminants will enter the organism’s body depends on the organism’s traits, such as feeding behavior [54]. If the contaminated NMPs do not enter the body, the bioavailability may be decreased, as NMP acts as a sink for the contaminant. In the case that contaminated NMP enters the body, the desorption rate is a defining factor. In earthworms, a low desorption rate of contaminants inside the body results in their elimination as the NMP is excreted [55,56]. On the other hand, the desorption of many contaminants has been observed, favored by the low gut pH [24,57]. For plants, it is more difficult to assess the desorption process; however, recent work has shown the desorption of heavy metals and PAHs from NMP in a simulated rhizosphere zone [58,59]. 

NMP may also present indirect effects on bioaccumulation. One interesting fact observed in the studies included in this review is that NMP can also affect the environmental behavior of contaminants by either increasing or decreasing, for example, porewater concentrations (i.e., bioavailable fractions). If NMP presence increases the porewater concentrations of a contaminant, it can result in a higher dermal/oral uptake in soil invertebrates or root uptake in plants of the contaminant from the medium, independent of the uptake of NMP by the organisms.

Finally, there are also mechanisms related to bioaccessibility, which is the contaminant fraction that crosses the biological barrier [60]. This means the translocation of the contaminant from the intestine to the lymph stream in oligochaetes and from the root surface to the root cell in plants. Bioaccessibility is influenced by the characteristics of the earthworm’s digestive system and the rhizosphere in plants, such as pH and microbiome composition. Recent studies show that NMP can alter gut and rhizosphere characteristics [61,62]. Therefore, it can be expected that NMP will also influence contaminants’ bioaccessibility. However, these mechanisms were not included or discussed in the publications. After crossing the biological barrier, the contaminant can take different metabolic pathways [63]. The contaminant may be metabolically available, which is the fraction that can potentially cause toxic effects. The contaminant may be stored in the cells in inert, non-toxic forms. Alternatively, the detoxification and elimination of the contaminant may take place. As a final reminder, it cannot be discarded that if the levels of NMP in the organism are high enough to cause toxic effects, it can also affect the overall bioaccumulation process. Organisms may lose the ability to eliminate contaminants when under stress. 

### 4.2. Knowledge Gaps and Limitations

Although there is an increasing number of publications on this topic, this systematic review identified several knowledge gaps. The first observation was the low number of different taxonomic groups studied. The terrestrial ecosystem comprises organisms living in different habitats and diverse traits. Particularly, there was a lack of studies with soil invertebrates other than oligochaetes.

Regarding the NMP characteristics, PS accounted for about 55% of the polymer type studied, and the spherical-shaped ones comprised almost 45%. Nevertheless, PE accounts for most plastic waste in the environment [64,65,66]. For NMP shape, fibers and irregular NMP are most abundant in the environment, and, in this review, only 10 studies have been performed to understand their potential in assisting in the bioaccumulation of contaminants. A similar observation was reported by Phuong et al. [67] that microspheres and beads are the most used in toxicity tests on marine organisms, but they do not represent the environmental scenario. While the internalization of perfectly shaped spherical NP has been shown for plants and earthworms, whether irregular-shaped NP would produce similar results remains to be answered. 

Toxicokinetic studies on chemicals comparing treatments with and without NMP were unavailable for soil organisms. Few publications assessed chemical uptake over time [34,41], and those results suggested that NMP might change the uptake and elimination rates of such contaminants. Toxicokinetic studies are important to allow the extrapolation of the effects in time and to higher biological levels [68]. Therefore, such studies should be encouraged. 

The evidence of NMP affecting bioaccumulation under a realistic scenario is limited. Extremely high NMP concentrations were used in many studies included in this review, and effects on contaminants’ bioaccumulation occurred mainly at ≥500 mg/kg. Testing high levels of NMP contamination is important to observe extreme exposure scenarios. However, in most studies, the effects on bioaccumulation were observed at unrealistically high concentrations (e.g., ≥10,000 mg/kg). Moreover, a high percentage of studies did not provide basic information on the NMP characteristics. For example, 21% of the publications reported herein did not inform the shape of the NMP used in their experiments. It is imperative for publications to include this information in the methodology section and to take it into account when discussing their results because the size and shape of NMP ultimately determine interaction and/or internalization by organisms. 

## 5. Conclusions

The main findings of this systematic review are synthesized below:

- Research is moving towards smaller particles, with an increasing number of publications using NP.
- In plants, most studies observed a decrease in contaminants’ bioaccumulation. In earthworms, on the other hand, an increase in contaminant bioaccumulation was observed in most of the experiments.
- In general, change in the bioavailable forms of the contaminants, either water or soil, was a major reason for the NMP to affect the contaminants’ bioaccumulation. This was valid for both plants and earthworms, even though the route of exposure differs between these two taxonomic groups.
- Little consistency was found regarding NMP characteristics. This could be caused by the specific relation between NMP characteristics and the overall study design (from spiking methodology to contaminant used and its concentrations). Still, this topic should be better explored. For example, there is still inconsistency among studies on the NMP size that can enter the plant root.
- Effects on bioaccumulation were more visible at high NMP concentrations, with few exceptions using realistic low concentrations. Future studies should focus on environmentally realistic concentrations.

## Data Availability

Not applicable.

## Supplementary Materials

The following supporting information can be downloaded at: https://www.mdpi.com/article/10.3390/toxics11020154/s1, Table S1: List of studies included in the systematic review.
