# Supplementary material for: A Systematic Review of Nano- and Microplastic (NMP) Influence on the Bioaccumulation of Environmental Contaminants: Part I—Soil Organisms
Source: Toxics. 2023 Feb 7;11(2):154. doi: 10.3390/toxics11020154 (PMC9958926; doi:10.3390/toxics11020154)
Supplement: Supplementary file 1 [file toxics-11-00154-s001.zip › toxics-2153518-supplementary.pdf]

Supplement material

A systematic review of nano- and microplastic (NMP) influence on the bioaccumulation of environmental contaminants: Part I - soil organisms

Paula S. Tourinho[1], Susana Loureiro[2], Maria Pavlaki[2], Klará Anna Mocová[1] Fabianne Ribeiro[2]

[1]Department of Environmental Chemistry, Faculty of Environmental Technology, University of Chemistry and Technology Prague, Technická 5, 166 28, Prague 6, Czech Republic

[2]Department of Biology and CESAM, University of Aveiro, Campus Universitário de Santiago, 3810-193 Aveiro, Portugal

Table S1: List of the studies included in the systematic review.

	First author's name	Year	Journal	doi
1	Boughattas, I	2022	Journal of Hazardous Materials	10.1016/j.jhazmat.2021.127578
2	Dong, Y M	2020	Environmental Pollution	10.1016/j.envpol.2019.113892
3	Gao, M L	2021	Environmental Pollution	10.1016/j.envpol.2020.115870
4	Hodson, M E	2017	Environmental Science and Technology	10.1021/acs.est.7b00635
5	Huang, C D	2021	Science of the Total Environment	10.1016/j.scitotenv.2020.142042
6	Huang, D	2022	Chemosphere	10.1016/j.chemosphere.2022.134729
7	Jia, H	2022	Chemosphere	10.1016/j.chemosphere.2021.132576
8	Kim, D	2022	Journal of Hazardous Materials	10.1016/j.jhazmat.2022.129194
9	Li, M	2021	Science of the Total Environment	10.1016/j.scitotenv.2020.144037
10	Lian, J P	2020	Environmental Pollution	10.1016/j.envpol.2020.114498
11	Liu, S Q	2021	Chemosphere	10.1016/j.chemosphere.2021.130967
12	Liu, Y	2022	Chemosphere	10.1016/j.chemosphere.2022.134059
13	Ma, J	2022	Chemosphere	10.1016/j.chemosphere.2022.135023
14	Sobhani, Z	2021	Environmental Technology and Innovation	10.1016/j.eti.2021.101476
15	Sun, W	2021	Chemosphere	10.1016/j.chemosphere.2020.128171
16	Tourinho, P S	2021	Ecotoxicology	10.1007/s10646-021-02424-3
17	Wang, F L	2021	Science of the total environment	10.1016/j.scitotenv.2021.147133
18	Wang, F Y	2020	Chemosphere	10.1016/j.chemosphere.2020.126791
19	Wang, F Y	2020	Toxics	10.3390/TOXICS8020036
20	Wang, H.-T. T	2019	Environmental Pollution	10.1016/j.envpol.2019.04.054
21	Wang, J	2020	Environmental Science & Technology	10.1021/acs.est.0c03712
22	Wang, J	2019	Environmental Pollution	10.1016/j.envpol.2019.03.102
23	Xu, G	2021	Science of the Total Environment	10.1016/j.scitotenv.2021.147016
24	Xu, G H	2021	Journal of Hazardous Materials	10.1016/j.jhazmat.2020.123966

Table S1 (continued)

25	Yang, Y	2022	Science of the Total Environment	10.1016/j.scitotenv.2022.154848
26	Zhang, S	2022	Applied Soil Ecology	10.1016/j.apsoil.2022.104469
27	Zhou, Y F	2020	Journal of Hazardous Materials	10.1016/j.jhazmat.2020.122273
28	Zong, X Y	2021	Ecotoxicology and Environmental Safety	10.1016/j.ecoenv.2021.112217
